# The Side Effects of Imatinib

**DOI:** 10.4274/Tjh-2011.0018

**Published:** 2013-09-05

**Authors:** Elmas Uzer, Ali Ünal, M. Yavuz Köker, Suat Ali Doğan

**Affiliations:** 1 University of Erciyes - Division of Hematology, Kayseri, Turkey; 2 University of Erciyes - Department of Infectious Diseases and Clinical Microbiology , Kayseri, Turkey

A 43-year-old male patient was diagnosed with chronic myelogenous leukemia (CML), and hydroxyurea treatment was begun 3 months before consultation. Physical examination revealed the spleen 2 cm below the costal margin. The Philadelphia chromosome was positive. After hydroxyurea treatment, imatinib (Gleevec) was started. On the 15th day of imatinib treatment, the patient was admitted because of fever, diarrhea, and generalized rash. Informed consent was obtained.

Whole blood count showed a hemoglobin concentration of 10.5 g/L, white cell count of 23,000/mm^3^, and platelet count of 240,000/mm^3^. Imatinib treatment was stopped to see if these symptoms were related to the drug. After cessation of imatinib, the fever resolved and the skin lesions disappeared. Imatinib was changed to dasatinib. The patient takes dasatinib at 100 mg and the treatment is on-going.

A wide spectrum of dermatologic toxicities has been associated with imatinib, among which a maculopapular rash is the most common event. Imatinib-induced skin rash is believed to be due to the blockade of the c-kit protein, which is present in skin. Severe toxicity of skin rash, fever, and diarrhea associated with imatinib has been reported. The initial dose for chronic-phase disease, 400 mg/day, is very well tolerated. A higher risk for developing rash will occur with imatinib doses higher than 600 mg/day, advanced age, and female sex. In many patients who experience unacceptable adverse effects, transient dose reduction or treatment interruption allows for patients to resolve the adverse effects.

## CONFLICT OF INTEREST STATEMENT

The authors of this paper have no conflicts of interest, including specific financial interests, relationships, and/ or affiliations relevant to the subject matter or materials included. 

